# Dealing with sexual boundary violation in mental healthcare institutions by government policies: the case of Flanders, Belgium

**DOI:** 10.1186/s12910-022-00778-9

**Published:** 2022-04-09

**Authors:** Lara Vesentini, Kim Dewilde, Frieda Matthys, Dirk De Wachter, Hubert Van Puyenbroeck, Johan Bilsen

**Affiliations:** 1 Mental Health and Wellbeing Research Group (MENT), Vrije Universiteit Brussel, Laarbeeklaan 103, 1090 Brussels, Belgium; 2 Universitair Psychiatrisch Centrum, Katholieke Universiteit Leuven, Leuven, Belgium

**Keywords:** Professional misconduct, Sexual boundary violation, Policy, Mental Health Care, Reporting, Survey

## Abstract

**Background:**

To prevent sexual boundary violations (SBV) in mental health care institutions overall governments require these institutions to report SBV incidents to a central registry and to develop institutional guidelines how to react. In Europe SBV policies are only recently developed or implemented, as is also the case in Flanders (Belgium). The implementation of a new institutional policy is always a challenge and can encounter resistance, especially when it concerns SBV, because they remain delicate and complex.

**Method:**

This study evaluated the extent to which mandatory policies on SBV have been implemented in mental health care institutions in Flanders, and possible factors for (non-)implementation of these policies. An online survey was sent to the executives of all mental health care institutions in Flanders (N = 162).

**Results:**

In total 56 executives of mental health care institutions filled out the survey (response rate 35%). Results showed that the implementation of an SBV policy in mental health care institutions is unfortunately inadequate and not all SBV incidents were reported to the central registry. Type of institution and opinions on the SBV policy were related to the (non-)implementation of the requirements.

**Conclusions:**

It is recommended that governments regularly communicate with mental health care institutions to better understand the concerns and difficulties concerning implementation of the required SBV policy and to support/stimulate an organisational culture of more openness and safety on this topic.

**Supplementary Information:**

The online version contains supplementary material available at 10.1186/s12910-022-00778-9.

## Background

Sexual Boundary Violations (SBV) are huge violations of human rights, especially in situations, such as in healthcare, where there is a disparity in power and status between the professional and the patient, and where the professional is seen as someone who can be totally trusted [1]. SBV in healthcare can be defined as any form of sexualized behavior committed within a professional role. It might include explicit sexual behavior such as (attempted) penetration or genital stimulation, as well as sexualized behavior in a broader sense, such as kissing, fondling, taking pictures of intimate body parts, presenting pornographic material, or sexualized remarks and (attempted) dating [1].

While SBV often takes place in a seemingly consensual way, ultimately it is often experienced by a patient as negative, unwanted or forced [1, 2]. Due to the emotional vulnerability of patients with mental problems, SBV within mental healthcare is even more precarious. Besides psychological consequences, such as feelings of shame, guilt and self-blame, the most salient aspect of SBV for involved patients is the abuse of their trust in situations where trust should be unconditional. The secure base is destroyed and without basic trust no effective therapy to help the client is possible [1, 3]. Findings, based on reporting of mental healthcare professionals who treated clients who did have sexual contact with previous mental healthcare professionals, suggest that about 75–90% of clients suffered harm, 1.8–11% of clients were hospitalized afterwards, 1–14% tried to commit suicide, and 0.3–1% committed suicide [4–6].

The prevalence of SBV is unclear. Studies based on surveys among mental healthcare professionals report that 1–7% of them started sexual relationships with their patients during their careers, and more male professionals reported this than their female colleagues [7–11]. Studies based on client-reported sexual relationships with their healthcare professional report rates between 0.2 and 2.2% [12, 13]. However, it cannot be ruled out that these percentages are an underestimation, as not all sexual relationships will be reported, due to response bias.

Some studies attempted to describe a profile of mental healthcare professionals that are involved in such sexual relationships [14, 15]. A common mentioned profile is the middle-aged male healthcare giver who is professionally isolated and currently undergoing some personal distress or midlife crisis (so called ‘love-sick therapist’). Although factors such as emotional distress may be contributing factors to SBV, there is no sound agreement that these factors are predictive of SBV [16–18].

How countries deal with SBV in healthcare institutions largely differs. In North America, Australia and New Zealand mandatory reporting of SBV is the rule, as well as protection for those who report such incidents. Furthermore, there are penal codes declaring SBV by professionals as a crime. In Europe, the implementation of laws and policies to deal with SBV has lagged about 20–25 years behind [1]. Nowadays, several European countries, including Flanders (Belgium), are also developing or recently developed policies on how to prevent SBV in healthcare institutions and how to deal with SBV when occurring. They mostly advise the formulation and implementation of clear guidelines in the institutions, as well as oblige the reporting of SBV incidents to a central registry. This reporting is considered as essential, because only when SBV incidents are known, a learning process can start. It contributes to more awareness and improvements in management and policy. Penal codes, declaring SBV of healthcare professionals to be a crime (punishment of these professionals with sexualized behavior towards a client) are still rather uncommon in Europe [1, 19–21].

To be successful in preventing SBV, such government policy measures must of course be adequately adopted by the healthcare institutions. However, it is not clear to what extent healthcare institutions are aware of the existence and concrete content of such mandatory government policy guidelines, neither to what extent they are prepared to accept and implement them. For example, we don’t know much about the attitudes of healthcare institution policy makers towards such measures, their perceived problems to implement them, or about their preparedness to officially report SBV incidents. After all, SBV incidents remain delicate and complex situations, not only evoking personal and group anxieties among healthcare workers but also possibly damaging the reputation of the institution itself. [22–26].

This study aims to investigate (1) the extent to which mandatory policies on SBV have been implemented in Mental Healthcare Institutions (MHCI), (2) knowledge about obligatory character of policies by the MHCI and opinions on such policies, and (3) possible factors related to (non-)implementation (e.g., type of MHCI, knowledge about obligatory character, opinions, presence of a reporting person). This will be done in Flanders (Belgium), as case where the government, as in other European countries, recently issued such SBV policy measures.

## Method

### Flemish context

The Flemish government (Belgium), more specifically the Flemish Agency of Care and Health (FACH), decided that from 2015 all accredited types of healthcare institution in Flanders would be obliged to implement an SBV policy, specifically requiring: (1) the development of a vision on how to deal with SBV in general, to be embedded in the institutional rules, (2) the development of a concrete procedure for how to react when an SBV incident occurs (the ‘reaction protocol’), (3) anonymous internal registration of suspected and confirmed SBV incidents, (4) official reporting of confirmed SBV incidents to the FACH, and (5) the appointment of a ‘reporting person’ in each MHCI, who ensures that the reaction protocol is followed. Although the development of an SBV policy is obligatory, institutions have the freedom to determine how the policy is fleshed out. A manual has been provided to help institutions to initiate their policy [21, 27, 28]. In this manual SBV was defined as every form of sexually oriented behavior, initiated by healthcare professionals, that is experienced by a client as negative, unwanted, or forced [28].

Currently, there is no penal code declaring SBV in healthcare as a crime. However, sexual behavior of the therapist can, in some cases, be considered as a criminal offense (e.g., criminal code regarding sexual assault or rape). In such cases there must be a lack of valid consent (i.e., the patient not giving consent to such behavior) and a certain degree of serious violations. Stroking the shoulders and hair, for example, is not considered to violate sexual integrity. Sometimes, this particular behavior is not a criminal offense, but ethical codes have been violated. In these cases, a report can be made to the professional association, and a disciplinary procedure can be initiated. This can lead to a warning or suspension of the therapist, or removal from the list of the professional association [29].

### Study design and study population

A cross-sectional study was conducted from 28 November 2018 until 25 January 2019. All different types of MHCI in Flanders (the Dutch-speaking northern part of Belgium), accredited by the FACH and with an adult patient population, were included in this study (N = 162): 19 mental health outpatient services (ambulatory mental healthcare), 27 psychiatric hospitals (long-term residential psychiatric care), 34 psychiatric departments of a general hospital (short-term residential psychiatric care), 27 psychiatric treatment homes (residential psychiatric care and living), 43 sheltered living services (residential assisted living), and 12 rehab centers for addiction (residential psychiatric care for persons with an addiction).

### Data collection

We retrieved from the FACH the contact details of all 162 executives of the accredited MHCI who were expected to have the most knowledge of or to be responsible for the implementation of the SBV policy at the MHCI. These were the general manager for mental healthcare services, psychiatric hospitals, and rehab centers for addiction, the general coordinator for psychiatric treatment homes and sheltered living services and the chief nurse for psychiatric departments of a general hospital. An e-mail with a link to an electronic survey was sent to these selected executives, followed by three e-mail reminders.

To compare the confirmed SBV incidents reported in the survey with the officially reported SBV incidents to the FACH (period 2016–2018), we also asked the FACH to send us the number of officially reports per type of MHCI.

### Questionnaire

The questionnaire (see Additional file 1) consisted of questions about (1) knowledge of obligations, (2) general opinions on SBV policy in MHCI (e.g., barriers, consequences, priorities) on an ordinal 5-point scale going from totally disagree to totally agree, (3) implementation of the specific SBV policy requirements (as described in the Flemish context), (4) actual occurrences of SBV incidents in the MHCI, and (5) type of MHCI. These questions were based on the Flemish government decree and the manual that was made available to help institutions start up their policy [21, 27, 28], previous literature on this topic [1, 24–26] and informal conversations with experts in the field. Furthermore, a pre-test was done among three executives, working in an MHCI with a patient population of minors, resulting in some minor, mainly textual adaptations to the questionnaire.

### Analysis

Frequencies and percentages are given to describe knowledge of the obligations, opinions on an SBV policy, implementation of the specific SBV policy requirements, actual occurrences of SBV incidents, and the type of MHCI. To investigate the association between, on the one hand, the implementation of the specific SBV policy requirements and, on the other hand, the type of institution, knowledge of obligations, opinions on SBV policy, and the presence of a reporting person, a two-tailed chi-square was used, or a fisher exact when appropriate. For the variable ‘opinions on SBV policy’, the ordinal 5-point scale was recoded into three options: ‘disagree’, ‘neutral’, and ‘agree’. IBM SPSS Statistics, version 23, was used for all analyses.

## Results

### Response rate

In total 56 executives of MHCI filled out the online survey (response rate 34.57%): 42.1% (n = 8) of all mental health outpatient services, 59.3% (n = 16) of psychiatric hospitals, 26.5% (n = 9) of psychiatric departments of a general hospital, 11.1% (n = 3) of psychiatric treatment homes, 20.9% (n = 9) of sheltered living services, and 91.7% (n = 11) of rehab centers for addiction.

### Knowledge about the obligatory character of the SBV measures

The majority of the responding executive staff in MHCI (80.4%) did know that they were obliged to develop an SBV policy, although a fifth was not. More than half of the executive staff knew that officially reporting SBV to the FACH (64.3%) was mandatory. Further analysis revealed that at the psychiatric departments of a general hospital (respectively 55.6% and 55.6%) and rehab centers for addiction (respectively 63.6% and 27.3%) this knowledge was lower compared to the other MHCI (not significant).

### Opinions on SBV policy

As shown in Table 1, a third of the executive staff members (33.9%) thought (agreed or totally agreed) that power imbalances between colleagues were a barrier to making official reports to the FACH. One out of seven responding executives thought a report was not made to avoid the visit of the FACH inspection (14.3%) and that it is better to organize an internal dialogue among the parties involved than follow a protocol (14.3%). Furthermore, 69.7% agreed that a reporting person lowers the barrier to discussing SBV. Further analysis revealed that there was no significant association between type of institution and opinion on SBV policy (not in table). Although not significant, it is of interest that more than half the responding executives of psychiatric departments of a general hospital (55.6%) agreed that power imbalances between colleagues is a barrier to reporting and 33.3% agreed there is little interest from the government concerning the need for an SBV policy. The majority of the executive staff members of psychiatric hospitals disagreed that an inspection is avoided by not reporting to the FACH (93.8%).

**Table 1:** Opinions on SBV policy (N = 56)

Opinions	Totally disagree		Disagree		Neutral		Agree		Totally agree	
	N	%	N	%	N	%	N	%	N	%
Power imbalances between colleagues are a barrier to reporting to the FACH	7	12.5	9	16.1	21	37.5	16	28.6	3	5.4
By not reporting to the FACH, an inspection is avoided	24	42.9	11	19.6	13	23.2	4	7.1	4	7.1
Little incidents are needlessly amplified when reporting to the FACH	16	28.6	21	37.5	13	23.2	5	8.9	1	1.8
Official reports to the FACH about a colleague will lead to a negative atmosphere	12	21.4	15	26.8	18	32.1	11	19.6	0	0
It is better to organize an internal dialogue among the parties involved than to follow a protocol	13	23.2	18	32.1	17	30.4	6	10.7	2	3.6
Other projects demand more priority (given the work pressure and limited time available)	25	44.6	19	33.9	8	14.3	3	5.4	1	1.8
There is little interest from the government in the need for an SBV policy	10	17.9	16	28.6	20	35.7	7	12.5	3	5.4
The development of an SBV policy is stimulated by former SBV incidents	1	1.8	9	16.1	12	21.4	23	41.1	11	19.6
A ‘reporting person’ lowers the barrier for discussing SBV	1	1.8	3	5.4	13	23.2	24	42.9	15	26.8

### Implementation of specific SBV policy requirements

More than half of the responding executives stated that the MHCI developed a reaction protocol (58.9%). In 28.6% of the MHCI there was an internal system to register suspected SBV incidents and in 44.6% MHCI there was such a system to register confirmed SBV incidents. Almost half of the MHCI (48.2%) had the policy of officially reporting confirmed SBV incidents to the FACH and in 58.9% MHCI a reporting person was present (Table 2). Of those MHCI where no reporting person was present (n = 23), almost all could report their suspicion or concern to one or more persons or services in the MHCI: ombudsman’s office, prevention and protection service at work, director, immediate superior or confidant.

**Table 2:** Implementation of specific SBV policy requirements (N = 56)

	1	2	3	4	5	6	7
	Vision	Institutional rules	Reaction protocol	Registration Suspected SBV	Registration Confirmed SBV	Official reports	Reporting person
Total MHCI where policy is present							
N	32	21	33	16	26	27	33
%	57.1	37.5	58.9	28.6	44.6	48.2	58.9
Type of MHCI	%	%	%	%	%	%	%
Psychiatric hospital (n = 16)	93.8	37.5	93.8	50	56.3	81.3	75
Psychiatric department of a general hospital (n = 9)	22.2	22.2	22.2	11.1	11.1	11.1	44.4
Psychiatric treatment home (n = 3)	66.7	66.7	66.7	0	66.7	66.7	100
Mental health outpatient service (n = 8)	62.5	25	62.5	12.5	25	37.5	37.5
Sheltered living services (n = 9)	55.6	55.6	55.6	22.2	44.4	55.6	55.6
Rehab center for addiction (n = 11)	27.3	36.4	36.4	36.4	63.6	27.3	54.6
*P-value*	0.004	0.582	0.008	0.176	0.135	0.011	0.285
Knowledge of obligations^a^	%	%	%	%	%	%	%
Obliged to develop SBV policy							
Yes (n = 45)	66.7	40	68.9	31.1	48.9	57.8	64.4
No (n = 11)	18.2	27.3	18.2	18.2	27.3	9.1	36.4
*P-value*	0.006	0.508	0.004	0.483	0.312	0.006	0.170
Officially reporting SBV incidents to the FACH							
Yes (n = 36)	66.7	47.2	72.2	33.3	50	61.1	61.1
No (n = 20)	40	20	35	20	35	25	55
*P-value*	0.090	0.051	0.011	0.365	0.401	0.013	0.779
Presence of a reporting person	%	%	%	%	%	%	n/a
Yes (n = 33)	72.7	45.5	69.7	39.4	54.4	54.5	
No (n = 23)	34.8	26.1	43.5	13	30.4	39.1	
*P-value*	0.007	0.170	0.060	0.039	0.103	0.289	

Specific SBV policy requirements: 1 = Vision about how to deal with SBV; 2 = SBV vision is embedded in the institutional rules; 3 = Reaction protocol; 4 = Registration of suspected SBV incidents in internal system; 5 = Registration of confirmed SBV incidents in internal system; 6 = Officially report confirmed SBV incidents to the FACH; 7 = Presence of a reporting person

^a^Fisher Exact

A minority (12%) of the MHCI would suspend the healthcare professional when SBV is suspected, but would never fire him/her, whereas when SBV is confirmed 39.3% MHCI would suspend and 53.6% would fire this healthcare professional.

### Factors related to (non-)implementaton of policy requirements

#### Type of MHCI

Significant differences were found based on type of institution, regarding the development of a vision about how to deal with SBV (*p* = 0.004), a reaction protocol (*p* = 0.008) and officially reporting of SBV incidents to the FACH (*p* = 0.011). Notably, the great majority of psychiatric hospitals have complied with these SBV policy requirements, but only a minority of psychiatric departments of a general hospital have done so (Table 2).

#### Knowledge of obligatory character

MHCI where executives did know developing an SBV policy and/or officially report SBV incidents to the FACH is mandatory significantly more often complied to the SBV policy requirements than MHCI where executives did not know this (Table 2). They more often developed a reaction protocol and more often had the policy of officially reporting these incidents if they would occur.

#### Opinions on SBV policy

MHCI where executive staff members did agree power imbalances between colleagues are a barrier to reporting less often had an internal system to register confirmed SBV incidents than MHCI where executives did not agree (neutral or disagree) (*p* = 0.039). MHCI where executives did agree that FACH inspections are avoided by not reporting to the FACH less often had an internal system to register suspected (*p* = 0.038) and confirmed (*p* = 0.010) SBV incidents, a policy to officially report SBV incidents (*p* = 0.018) or a reporting person (*p* = 0.010). MHCI where executives did agree that it is better to organize an internal dialogue among parties involved than follow a protocol develop a vision about how to deal with SBV less often (*p* = 0.039). MHCI where executives did agree or were neutral that a reporting person lowers the barrier to discuss SBV developed a vision about how to deal with SBV (*p* = 0.046) and a reaction protocol (*p* = 0.045) more often (not in table).

#### Presence of a reporting person

When a reporting person was present in an MHCI, the MHCI had more often developed a vision about how to deal with SBV (*p* = 0.007) and an internal system to register suspected SBV incidents (*p* = 0.039) (Table 2).

### Occurrence of SBV incidents in MHCI

In the past three years (period 2016 till 2018), in 22 of the 56 questioned MHCI there were 53 suspected SBV incidents, and in 18 of the MHCI there were 30 confirmed SBV incidents (Table 3). Most confirmed SBV incidents were in psychiatric hospitals (n = 12) and rehab centers for addiction (n = 12). When comparing these unofficially confirmed SBV incidents to the official reported incidents, as was requested by the FACH, especially the underreporting in the psychiatric hospitals is noticed (12 reports versus 9 reports).

**Table 3:** Occurrence of SBV incidents in MHCI (period 2016–2018)

Type of MHCI	Suspected SBV incidents		Confirmed SBV incidents		Official reports as known by the FACH
	N cases	N MHCI	N cases	N MHCI	N cases
Psychiatric hospital	20	9	12	7	9
Psychiatric department of a general hospital	5	1	1	1	n/a
Psychiatric treatment home	0	0	1	1	1
Mental health outpatient service	3	3	1	1	1
Sheltered living services	6	2	3	2	2
Rehab centers for addiction	19	7	12	6	n/a
Total	53	22	30	18	

n/a means that at time of the study the official records for the psychiatric departments of a general hospital and rehab centers for addiction were not known or registered

## Discussion

This study shows that the knowledge about and implementation of an SBV policy in Flemish MHCI, as required by the FACH since 2015, is unfortunately inadequate. Around 20% of the executives do not know that the development of an SBV policy is mandatory and even 36% do not realize that official reporting of SBV incidents to the FACH is obliged. About 40% of the MHCI had not developed a vision and reaction protocol; more than half of the MHCI had no internal registration system for suspected (71%) or confirmed (55%) SBV incidents as is required. In 30% of the MHCI no reporting person was assigned and finally not all SBV incidents were reported to the FACH. The type of institution, knowledge of obligations and opinions on SBV policy were related to the implementation of these requirements. Overall, psychiatric hospitals implemented specific SBV policy requirements far more often than psychiatric departments of a general hospital. Knowledge of the obligations was positively associated with the implementation of the requirements. MHCI where executives with the opinion that an inspection could be avoided by not reporting to the FACH complied less often with the specific SBV policy requirements than those MHCI that did not share this opinion. These findings of the case in Flanders will probably also be interesting for policymakers in other countries with similar approaches towards SBV in mental healthcare.

An important finding is that the institutions insufficiently are aware and acknowledge the relevancy of such SBV policy. In Flanders, about a third of the executives do not know that the implementation of an SBV policy and reporting of SBV incidents to the FACH is mandatory. In addition, about a fifth found that other projects demand more priority. This indicates that the communication about this SBV policy, its obligatory character and its importance is far from optimal. Possibly this request to implement an SBV policy is overlooked due to an overload of incoming information and administrative demands from the authorities. The lack of clear (financial) implementation incentives, as well as control and compelling negative consequences (e.g., loss of accreditation) in case of not fulfilling the requirements, will also not stimulate MHCI executives to comply to the requested measures. However, perhaps the most important condition for successfully implementing a policy is to develop such policy with stakeholders and key figures from a participatory approach (and not imposed from above), taking into account e.g., the MHCI culture, implementation climate, self-efficacy of personnel [30, 31].

Perhaps enforced by the non-participatory approach, also fear of the consequences for interpersonal relations on the work floor as well as insecure reactions from authorities seems to play an impeding role to meet the guidelines in this study. Some MHCI in Flanders clearly state to prefer internal protocols and solutions when confronted with SBV, over following the authority’s protocol, e.g., via internal dialogue between involved parties. One out of seven executives thought that often SBV incidents would not be officially reported to avoid visits by the FACH inspection. One out of three executives found that power imbalances between colleagues might increase the reluctance to report, pointing to fear of possible retaliation by the involved colleague, in line with findings of previous papers [1, 22, 24–26, 32–34]. For the mandatory reporting to function (i.e., actually reporting), several issues should be taken into account [35]. A Work Group (of the American Psychiatric Association) proposes some guidelines in this regard. They emphasize the importance of maintaining the confidentiality of such reports, call for penalties when there is a failure to report, and they advocate immunity for reporting [36]. Furthermore, it is important that patients should determine whether a report is to be made, as some patients might be not willing to report [36, 37]. Another challenge concerns ‘third-party information’ (i.e., when this information about an SBV incident is indirectly obtained, e.g., via disclosure of a patient), which is sometimes difficult to rely on, and there is the risk of compromising professional secrecy. Additionally, it should be determined whether there is still a current and ongoing risk of harm from this healthcare professional that has engaged in such misconduct [35, 37].

Installing a central function within the institution, in the form of a ‘reporting person’ that employees can contact in case of questions and problems related to SBV, is often seen as an important step in the prevention of SBV incidents [1, 26, 28]. The results of this study also points to that direction. Most respondents confirmed it stimulated discussions about SBV and MHCI with such function had also more often a registration system for suspected SBV incidents. However, it did not increase the actual registering and reporting of cases, which probably again relates to worries about possible consequences, as mentioned earlier.

Striking in our results was the underrepresentation in this survey of psychiatric departments in general hospitals, and the fact that these also had implemented SBV policy requirements less often than the other MHCI. Explanations for this are not clear. Possibly, (participating in studies about) SBV measurements specific meant for mental healthcare institutions, are regarded as even less important by the executives of these-often large-hospitals, with so many other protocols and whose core business lies in the many other medical departments.

The strength of this study is that it provides some plausible explanations for the finding that several MHCI had not complied with the requests of the government in implementing and following guidelines about SBV, therefore giving indications for improvement, probably also applicable in other countries in similar processes. A limitation of the study is the rather low response rate, so selection bias cannot be excluded. Possibly MHCI where no SBV policy was implemented or where it was not perceived as an important topic were possibly less willing to participate in this study. Another limitation is the low total number of MHCI, which is of influence on the statistical power [38]. Furthermore, it cannot be excluded that socially desirable answers were given. Finally, the results on opinions on SBV policy should be interpreted with caution. It cannot be excluded that the questions about opinions might be considered to be somewhat suggestive. In addition, it should be taken into account that the opinion of executives is not necessarily representative of the opinion of all healthcare providers. Furthermore, the opinions of executives do not necessarily apply to their own MHCI, as this is not formulated as such in the questionnaire.

## Conclusions

This study shows that several MHCI don’t know the content and obligatory character of SBV policy requirements as prescribed by their Government, or worry about the consequences when implementing such policy. Clearer communication by the authorities about the content of the policy requests, their mandatory character, regular monitoring and possible consequences when requirements are not followed, is therefore advisable. Financial or educational incentives can be useful in promoting the implementation of an SBV policy. Apparently, this is even more relevant for psychiatric departments embedded in a larger organizational structure (like a general hospital). To enhance reporting of SBV incidents to a central registry, anonymity of involved healthcare professionals must be guaranteed and concrete support and guidance in how to prevent such events in the future should discretely be discussed with(in) the involved MHCI. Regardless the occurrence of such events, professionals should ideally be offered, voluntarily, on a continuous basis and integrated in their daily work, training and support about sexual feelings towards clients, about boundaries, identify personal risk situations and how to manage them. Last but not least, supporting and stimulating an organisational culture of more openness and safety on this topic is of utmost importance to a succesfull implementation of a meaningfull SBV policy.

## Supplementary Information

**Additional file 1**: Survey

## Data Availability

The dataset used and analyzed during the current study is not publicly available due to containing information that could compromise the privacy of mental health care institutions but is available from the corresponding author on reasonable request.
