# Supplementary material for: Dealing with sexual boundary violation in mental healthcare institutions by government policies: the case of Flanders, Belgium
Source: BMC Med Ethics. 2022 Apr 9;23:40. doi: 10.1186/s12910-022-00778-9 (PMC8994893; doi:10.1186/s12910-022-00778-9)
Supplement: Supplementary file 1 — Additional file 1: Survey [file 12910_2022_778_MOESM1_ESM.docx]

**Additional files**

**Additional file 1: Survey**

Survey questions concerning policy about sexual boundary violation in mental healthcare institutions (MHCI)

Type of MHCI

**To which group of mental healthcare institution does your institute belong?**

- Psychiatric hospital
- Psychiatric department of a general hospital
- Psychiatric treatment home
- Mental health outpatient service
- Sheltered living services
- Rehab center for addiction

**In which province is your institute located?**

- West Flanders
- East Flanders
- Flemish Brabant
- Antwerp
- Limburg

Knowledge of obligations

**Indicate in the statements below which you think are true or false.**

|  | True | False |
| --- | --- | --- |
| 1. An SBV incident in a healthcare institution must always be reported to the Flemish Agency of Care and Health (FACH)* |  |  |
| 2. There is a colleague in my institution who is known to have had a relationship with a patient |  |  |
| 3. According to the Flemish government an SBV incident can be registered as the institution wishes. For example, via a note in the personnel file |  |  |
| 4. The government oblige healthcare institutions to develop an SBV policy* |  |  |
| 5. Within my institution an employee has already been fired because of SBV |  |  |

*Note*. *Only statement 1 and 4 are relevant to determine whether MHCI know about the obligatory character of these SBV measurements, and therefore only these statements are discussed in the manuscript.

Opinions on SBV policy

**Please indicate in the list below to what extent you agree with these statements.**

*(1= totally disagree, 2= disagree, 3= neutral, 4=agree, and 5=totally agree)*

|  | 1 | 2 | 3 | 4 | 5 |
| --- | --- | --- | --- | --- | --- |
| Power imbalances between colleagues are a barrier to reporting to the FACH |  |  |  |  |  |
| By not reporting to the FACH, an inspection is avoided |  |  |  |  |  |
| Little incidents are needlessly amplified when reporting to the FACH |  |  |  |  |  |
| Official reports to the FACH about a colleague will lead to a negative atmosphere |  |  |  |  |  |
| It is better to organize an internal dialogue among the parties involved than to follow a protocol |  |  |  |  |  |
| Other projects demand more priority (given the work pressure and limited time available) |  |  |  |  |  |
| There is little interest from the government in the need for an SBV policy |  |  |  |  |  |
| The development of an SBV policy is stimulated by former SBV incidents |  |  |  |  |  |
| A ‘reporting person’ lowers the barrier for discussing SBV |  |  |  |  |  |

Implementation of specific SBV policy requirements

**How is attention paid to SBV within your institution?**

*Select all options*

- There is a vision on how to deal with suspected and confirmed SBV incidents*
- How to deal with SBV is embedded in the institutional rules*
- There is a document (reaction protocol) that indicates how to act as institution when SBV incidents occur*
- SBV is a theme in the internal manual on quality
- Internal efforts are made to promote an open communicative climate
- Other, ……

*Note.* *Only the first three options summed up here are SBV policy requirements, and therefore only these options are discussed in the manuscript.

**What particular actions are taken when SBV incidents are suspecter or confirmed?**

*Please tick what applies within your institution.*

|  | In case of suspected SBV incidents | In case of confirmed SBV incidents |
| --- | --- | --- |
| 1. A note will be made in the personnel file and/or patient file |  |  |
| 2. A registration will be made in internal system* |  |  |
| 3. A report will be made to the FACH* |  |  |
| 4. A report will be made to the police / prosecutor |  |  |

*Note.* *Only action 2 (both in case of suspected and confirmed SBV incidents) and action 3 (only in case of confirmed SBV incidents) are SBV policy requirements, and therefore only these actions are discussed in the manuscript.

**Who can employees contact with suspicions or concerns about SBV in your institution?**

*Select all options*

- An officially appointed person (reporting person)*
- Ombudsman service
- Service on prevention and protection at work
- No one / no service
- Other

*Note.* *Only the first option (i.e., the presence of a reporting person) is an SBV policy requirement, and therefore only this option is discussed in the manuscript.

Actual occurrences of SBV incidents

**Have there been *suspected* SBV incidents in your institution?** *If yes, give the number of these incidents of the past 3 years.*

Number: …

**Have there been *confirmed* SBV incidents in your institution?** *If yes, give the number of these incidents of the past 3 years.*

Number: …

Ending

**Do you have other remarks you want to share with us?**

Answer: …….

Thank you
